# Localization of TCR-expressing natural intraepithelial lymphocytes in the colon epithelium depends on GPR15 and epithelium-derived C10ORF99

**DOI:** 10.1016/j.mucimm.2026.100355

**Published:** 2026-06-01

**Authors:** Gerald J. O’Connor, Jihae C. Choi, Nguyen T. Van, Anthony M. Klutkoski, Lucia C. Robertson, Sangwon V. Kim

**Affiliations:** a Department of Microbiology and Immunology, Sidney Kimmel Medical College, Thomas Jefferson University, Philadelphia, PA, USA; b Sidney Kimmel Cancer Center, Jefferson Health, Philadelphia, PA, USA

**Keywords:** C10ORF99, GPR15L, GPR15LG, GPR15, Intraepithelial lymphocytes, Migration, Large intestine

## Abstract

Intraepithelial lymphocytes (IELs) are among the largest lymphocyte populations in the body and play a crucial role in maintaining the integrity of epithelial barriers at mucosal sites, which are highly immunostimulatory. Therefore, understanding how these cells are generated, localized within the epithelium, and contribute to barrier immunity is essential. Although most research on IEL biology has focused on the small intestine, IELs are also present in the large intestinal epithelium. Additionally, the homing receptor-ligand pairs that regulate T cell localization in the lamina propria beneath the epithelium differ between the small and large intestines: CCL25-CCR9 in the small intestine and C10ORF99-GPR15 in the large intestine. CCL25-CCR9 is also necessary for proper localization of IELs in the small intestine, but the mechanisms controlling IEL localization in the large intestine remain unknown. Here, we demonstrate that the C10ORF99-GPR15 pathway is crucial for the presence of TCR + natural IELs in the large intestinal epithelium, a process that requires epithelium-derived C10ORF99 (GPR15L).

## Introduction

The highly immunostimulatory intestinal lumen is separated from the underlying tissue by a single layer of epithelial cells. Disruptions in this barrier can allow luminal antigens and microbes to cross it. It leads to inflammation, which, if left uncontrolled, can lead to inflammatory bowel disease (IBD)^1,2^. To help maintain homeostasis as a barrier, the epithelium is also equipped with a specialized cell population known as intraepithelial lymphocytes (IELs)^3–7^. While these cells are generally considered to be unconventional lymphocytes, they actually make up one of the most abundant lymphocyte populations in the body^3,4,8^. These cells permanently reside in the epithelium and survey for any perturbations of the epithelial cells^3,6^. Additionally, while IELs have cytotoxic potential^6^, they also have a high activation threshold^9^ and can dampen inflammation^10^. This allows IELs to be responsive to infected or transformed epithelial cells, but also capable of existing in a highly immune-stimulatory environment without causing excessive inflammation.

IELs can be divided into several categories. While many IELs are T cells, there are also T cell receptor (TCR)-negative IELs, such as B cells and innate lymphoid cells^3,4^. TCR^+^ IELs can be categorized according to developmental pathways as natural IELs (nIELs) or peripherally induced IELs (pIELs)^3,4,7^. nIELs migrate directly from the thymus to the intestinal epithelium, whereas pIELs are conventional αβ T cells that migrate to the epithelium after activation^3,4,6,7^. These nIELs, which migrate directly from the thymus, are also known as recent thymic emigrants and can migrate directly from the blood to local tissues, such as the small intestine^11^. Both TCR^+^ nIELs and pIELs appear to have limited TCR diversity^3^; however, this is more pronounced in nIELs^3,8^. Additionally, unlike pIELs, nIELs are not MHC-restricted and may bind glycolipids and other metabolites or small peptides, as well as self-antigens^3,12,13^. These nIELs also express receptors commonly associated with NK cells^3,8^. Interestingly, pIEL and nIEL TCR repertoires do not overlap, indicating distinct precursors and potentially distinct functions within the intestines^3^.

The large intestine contains more microbiota and T regulatory cells than the small intestine^14–16^. Additionally, the small and large intestines use distinct homing receptors to recruit lymphocytes to the lamina propria beneath the epithelium^17^. In the small intestine, lymphocyte migration is regulated by the chemokine receptor CCR9 and its ligand CCL25^17–19^. However, T cell migration to the large intestine depends on the homing receptor GPR15 and its ligand, C10ORF99 (GPR15L, GPR15LG)^20,21^. GPR15 is a class A GPCR initially discovered as a coreceptor for HIV^22,23^. It has been identified later as a homing receptor for the large intestine^20^. It controls the trafficking of both T effectors and T regulatory cells to the large intestine lamina propria^20,24^. GPR15 was de-orphaned with the discovery of C10ORF99 (GPR15LG, GPR15L) as a functional ligand^21,25,26^. In the small intestine, CD8^+^ recent thymic emigrants migrate to the epithelium via CCL25-CCR9 signaling^11^. However, it is unknown how nIEL migration is regulated in the large intestine. Additionally, while the C10ORF99-GPR15 signaling axis has been identified as an important mediator of T cell trafficking to the large intestine lamina propria^20,21^, it is unknown whether it is also required for the IEL localization in the large intestine epithelium.

Here, we investigated the role of the C10ORF99-GPR15 pathway in IEL localization to the large intestinal epithelium. We found that both C10ORF99 and GPR15 are required for the presence of TCR^+^ nIELs in the large intestinal epithelium; however, they are not required for TCR^+^ pIELs or TCR^−^ IELs. Additionally, we determined that epithelium-derived C10ORF99 is specifically required for the recruitment of TCR^+^ natural IELs but is only partially necessary for the recruitment of lamina propria T cells.

## Results

### GPR15 is required for the localization of TCR-expressing natural IELs to the epithelium of the large intestine

**Fig. 1.**
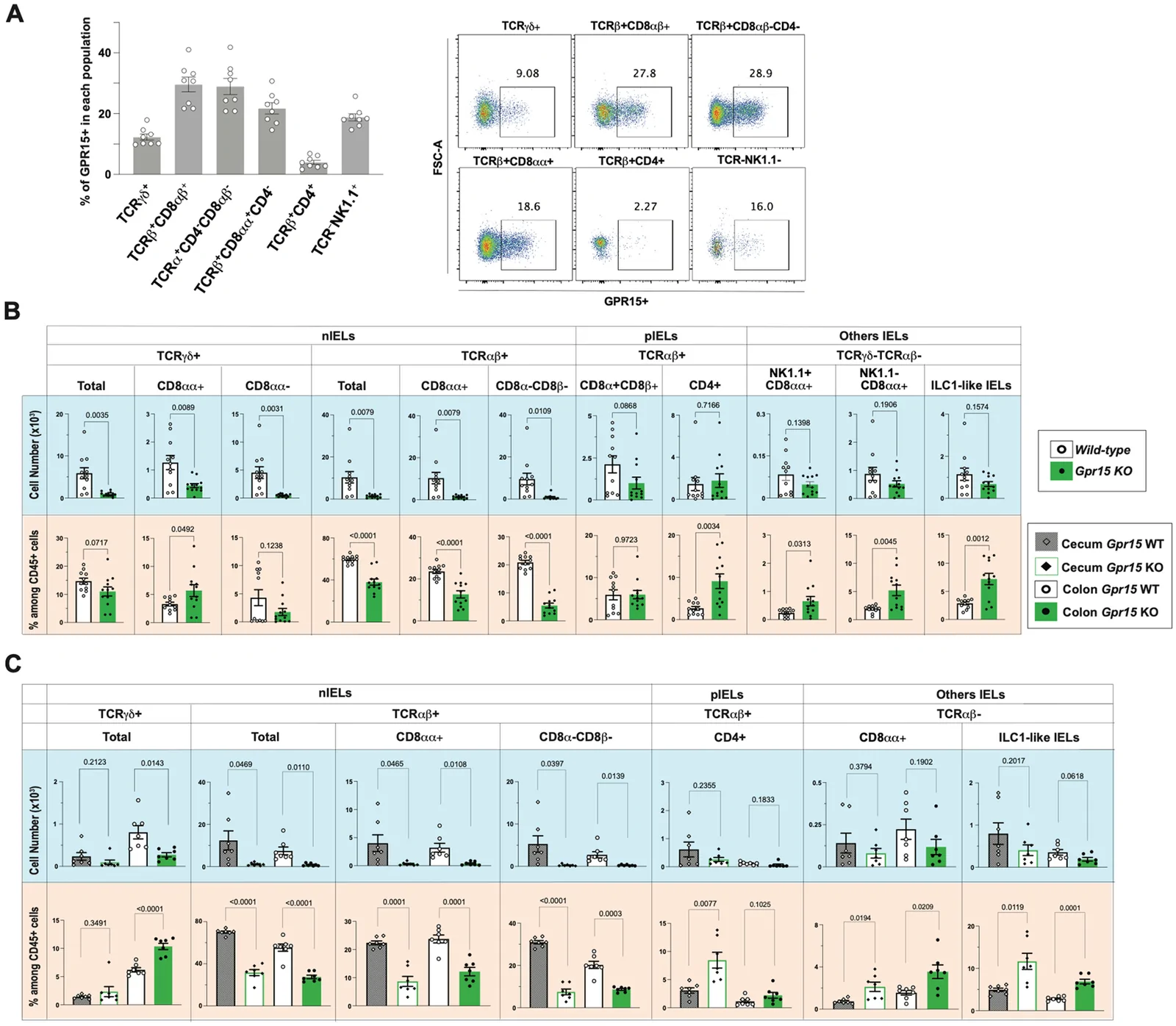
GPR15 is required for the recruitment of TCR-expressing natural IELs to the large intestine epithelium. (A) The percentage of GPR15 + cells in each IEL subset of the large intestinal epithelium is shown, along with representative flow cytometry plots (n = 8). (B) Cell number (top) and percentage among CD45^+^ cells (bottom) of IELs (nIELs, pIELs and other IELs) in the large intestine epithelium comparing wild-type vs *Gpr15* KO mice (n = 11 (WT), 12 (KO)). (C) Cell number (top) and percentage among CD45^+^ cells (bottom) of TCR + IELs in the epithelium of the cecum and the colon between wild-type vs *Gpr15* KO mice (n = 7 each). Data are shown as mean ± SEM. Populations compared using Student’s *t*-test. Each dot represents one mouse.

GPR15 has been identified as being necessary for T cell migration to the lamina propria of the large intestine^20^, and we recently demonstrated that C10ORF99 functions as a sole ligand of GPR15 in the large intestine lamina propria^21^. Considering that the CCR9-CCL25 pathway mediates T cell trafficking to both the lamina propria and the epithelium of the small intestine^11,27–29^, we hypothesized that the GPR15-C10ORF99 mediates IEL localization to the large intestine epithelium. We first determined whether IELs in the large intestine epithelium express GPR15 by flow cytometry (Fig. S1). We found that GPR15 expression levels on the cell surface vary depending on IEL subsets, with the lowest levels in TCRβ^+^CD4^+^ cells (Fig. 1A). Because that expression does not imply that GPR15 functions in those cells and because active engagement of GPR15 can lead to its downregulation on the cell surface, we further investigated TCR + and TCR- IEL populations in the absence of GPR15. We found that the TCR^+^ nIEL population was significantly reduced in the large intestine of *Gpr15* KO mice, with reductions in overall TCRαβ^+^ T cells as well as subsets like CD8αα^+^TCRαβ^+^ T cells and TCRγδ^+^ T cells (Fig. 1B). This decrease was not observed in CD4^+^TCRαβ^+^ or CD8αβ^+^TCRαβ^+^ cells, which are mostly pIELs^4^ (Fig. 1B). TCR^−^ IELs were also not affected by the absence of GPR15 (Fig. 1B).

To determine whether differences in IEL populations exist across different parts of the large intestine, we analyzed the cecum and colon in WT and *Gpr15 KO* mice. We observed similar trends between cecum and colon, with reduced TCR^+^ nIELs and minimal to no effect in TCR^+^ pIELs or TCR^−^ IELs in the absence of *Gpr15* (Fig. 1C). This is consistent with T cell trafficking to the large intestine lamina propria via C10ORF99-GPR15, which shows similar trends in proximal vs distal large intestine trafficking^21^. Overall, these results indicate that GPR15 is required for the localization of TCR-expressing natural IELs within the large intestine epithelium.

### C10ORF99 is required for the localization of TCR-expressing natural IELs to the large intestine epithelium

**Fig. 2.**
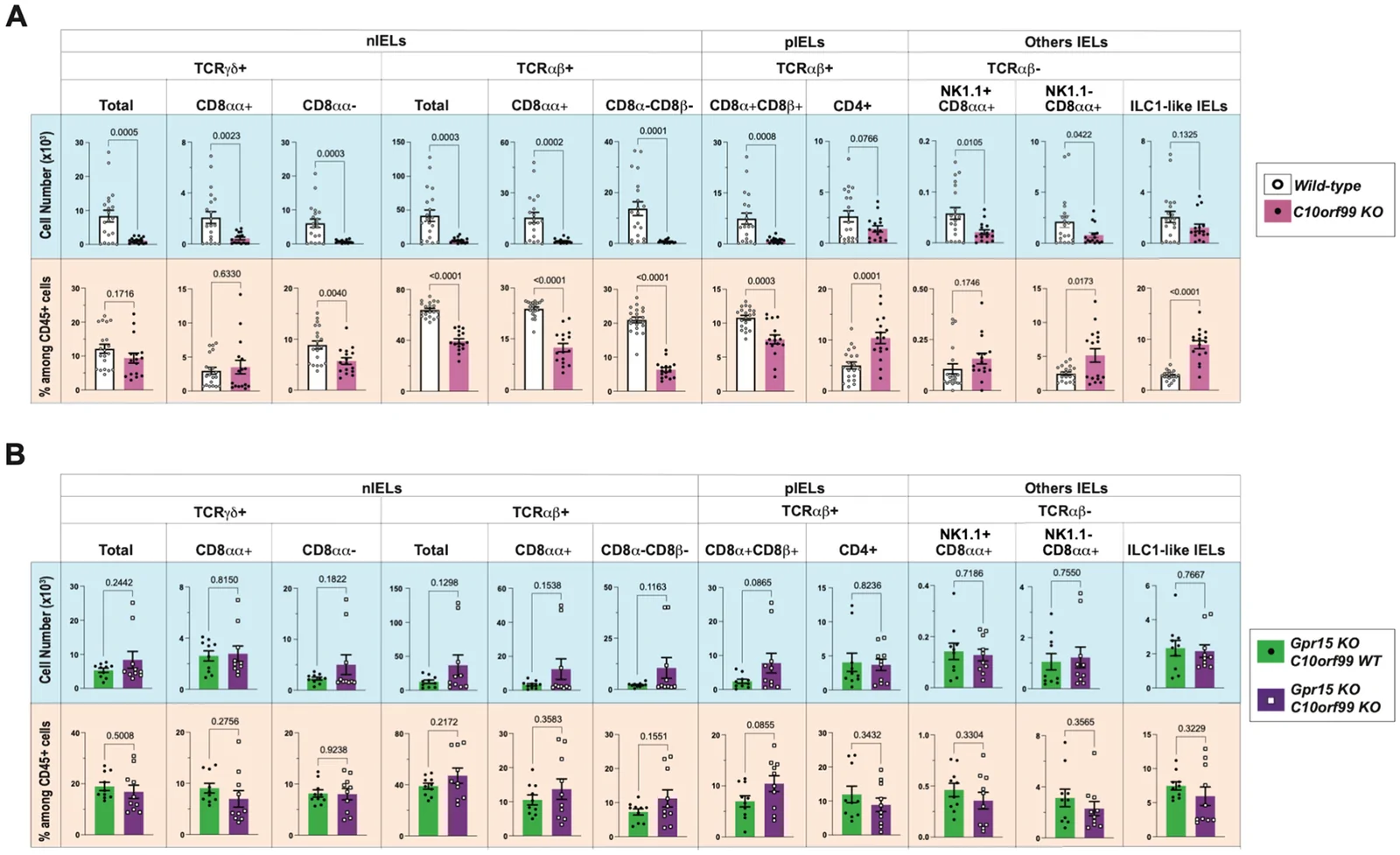
C10ORF99 mediates the recruitment of TCR-expressing natural IELs to the large intestine epithelium via GPR15. (A) Cell number (top) and percentage among CD45^+^ cells (bottom) of IELs in the large intestine epithelium comparing wild-type and *C10orf99* KO (pink) mice (n = 20 (WT), 16 (KO)). (B) Cell number (top) and percentage among CD45^+^ cells (bottom) of IELs in the large intestine comparing *Gpr15* KO (green) and *Gpr15/C10orf99* double KO mice (purple) (n = 10 each). Data are shown as mean ± SEM. Populations compared using Student’s *t*-test. Each dot represents one mouse.

Because GPR15 affects nIELs in the large intestine, we took the reciprocal approach and determined the effects of C10ORF99, the ligand for GPR15, using WT and *C10orf99* KO mice. We observed similar decreases in the TCR^+^ nIEL population in the absence of C10ORF99 (Fig. 2A), with no significant impact on CD4^+^TCR^+^ pIELs or TCR^−^ IELs (Fig. 2A). This indicates that the TCR^+^ nIEL population may rely on the C10ORF99-GPR15 signaling axis for their recruitment or retention within the large intestine epithelium. The only discrepancy between *Gpr15 KO* and *C10orf99* KO in IEL phenotype is a decrease of CD8αβ^+^TCRαβ^+^ pIELs in the large intestine of *C10orf99* KO mice. Notably, baseline levels of CD8αβ^+^TCRαβ^+^ pIELs varied across cohorts despite co-housing of experimental mice prior to analysis, and CD4^+^TCRαβ^+^ pIELs were uniformly low. These observations suggest that mice housed under specific pathogen-free (SPF) conditions may lack sufficient and consistent environmental antigen exposure to support full pIEL development, making the pIEL compartment variable and, most likely, accounting for this minor phenotypic difference. In addition, we saw no significant change in IEL presence within the small intestine when comparing WT mice, and *C10orf99 KO* mice (Fig. S2). This is consistent with evidence that *C10orf99* is not expressed in the small intestine^25^.

We have previously shown that C10ORF99 has both GPR15-dependent and GPR15-independent functions in the large intestine^21^. Therefore, we determined whether the effects of C10ORF99 on IELs that we observed in Fig. 2 were dependent on GPR15 by comparing the effects of C10ORF99 on IELs in the absence of GPR15 using *Gpr15 KO* and *Gpr15/C10orf99 double KO* mice. We found no significant GPR15-independent effect of C10ORF99 on TCR^+^ nIELs (Fig. 2B).

Together, these data reveal a previously unappreciated role of the C10ORF99-GPR15 pathway in the localization of intraepithelial lymphocytes specifically within the large intestine epithelium. This appears to regulate only TCR^+^ nIEL populations, not all IEL subsets.

### Generation of C10orf99 conditional KO mice

**Fig. 3.**
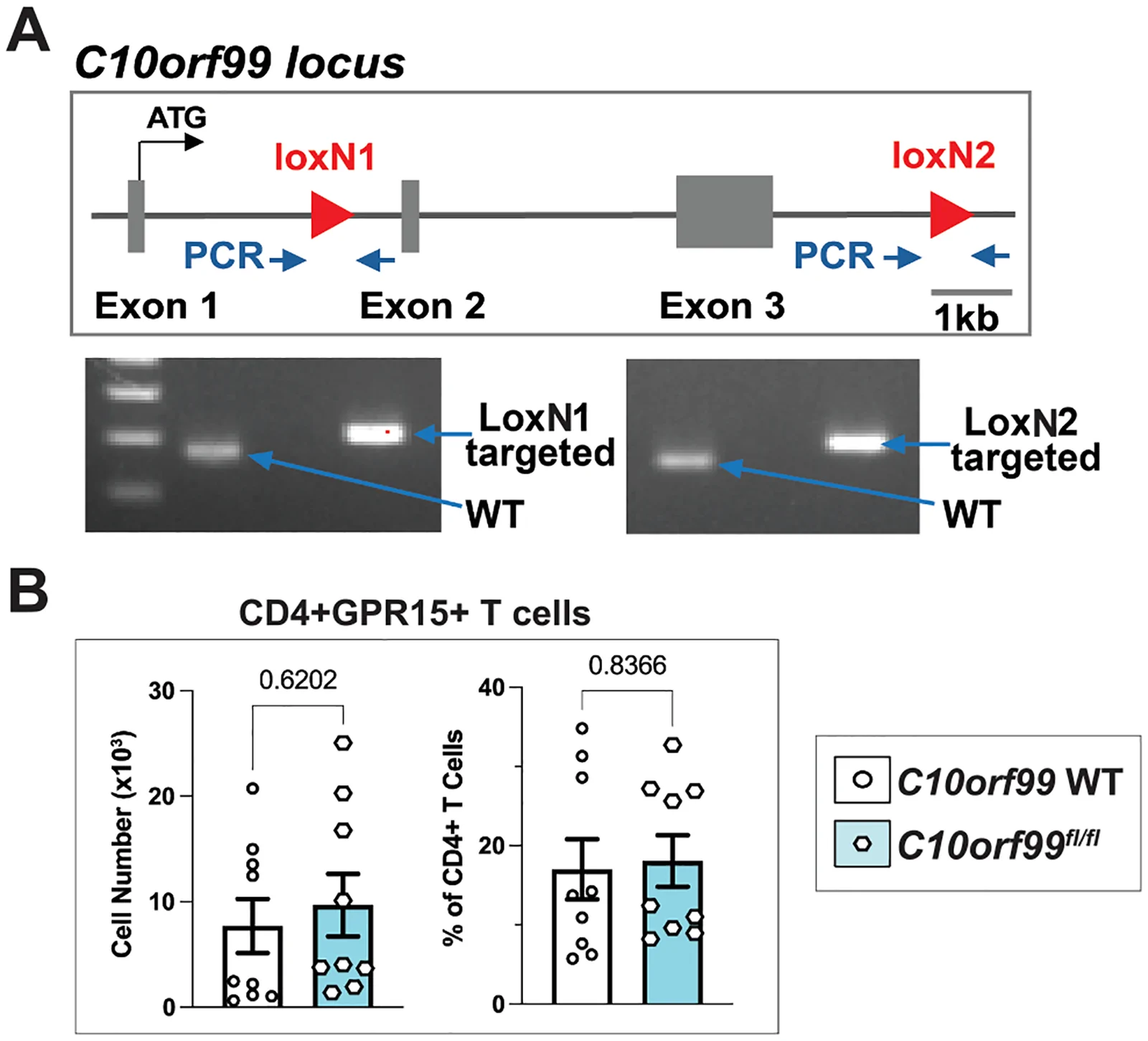
*C10orf99* conditional knockout generation. (A) *C10orf99* locus is shown with the location of two LoxN sites inserted. Insertion of each loxN site was verified by PCR (B). The number and the percentage of CD4 + GPR15 + cells were examined in wild-type and *C10orf99*^*fl/fl*^ mice (n = 9 each), and showed no difference, confirming that loxN insertions did not affect C10ORF99 expression. Data are shown as mean ± SEM. Populations compared using Student’s *t*-test. Each dot represents one mouse.

Under steady-state conditions, C10ORF99 is produced at least by both epithelial and endothelial cells of the large intestine^21^. Although extravasation of T cells or IEL precursors into the large intestine likely depends on C10ORF99 expressed by blood endothelial cells^21^, it remains unclear whether C10ORF99 guides these cells further toward the epithelium or is involved in their retention in the lamina propria. Considering a significant expression of C10ORF99 from colonic epithelium^21^, we decided to investigate the functional relevance of epithelium-derived C10ORF99 and generated *C10orf99* conditional knockout mice (*C10orf99 cKO)* for this purpose. We introduced loxN sites^30^ to the noncoding regions flanking exon 2–3 to generate a conditional knockout allele (Fig. 3A). After validating the addition of loxN sites through PCR (Fig. 3A), we determined if the loxN site additions influenced *C10orf99* expression and functionality by comparing CD4^+^ T cells and GPR15-expressing CD4^+^ T cells in WT and *C10orf99*^*fl/fl*^ mice. We did not observe any significant differences in GPR15-mediated trafficking of T cells to the lamina propria between the WT and *C10orf99*^*fl/fl*^ mice (Fig. 3B), indicating that C10ORF99 expression was not perturbed by the insertion of two loxN sites.

### Epithelium-derived C10ORF99 is required for the localization of TCR-expressing natural IELs

**Fig. 4.**
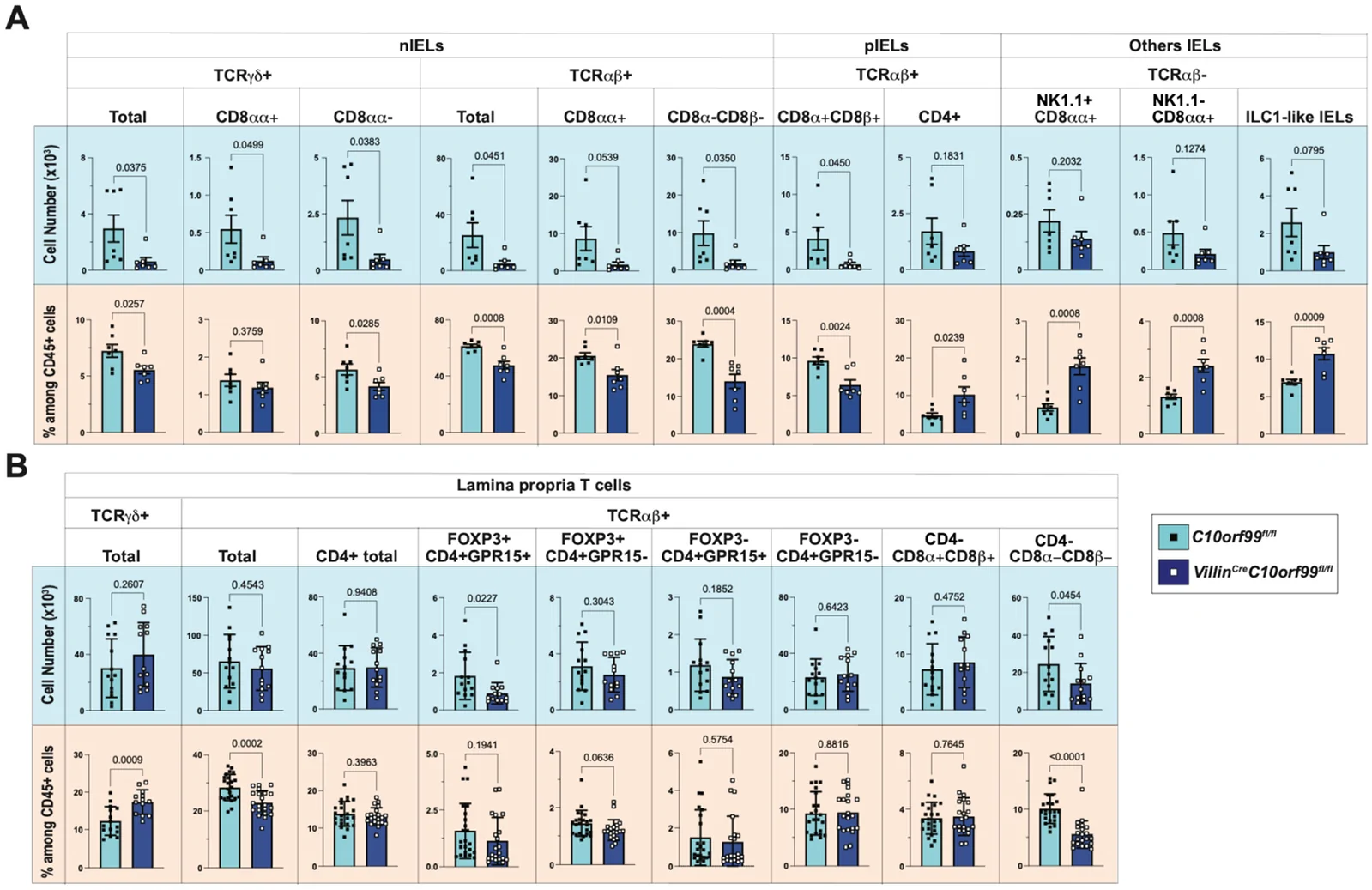
Epithelium-derived C10ORF99 is required for the localization of TCR-expressing natural IELs in the large intestine epithelium. (A) Cell number (top) and percentage of CD45 + cells (bottom) of TCR + IELs in the large intestine epithelium comparing *C10orf99*^*fl/fl*^ vs. *Villin*^*Cre*^*C10orf99*^*fl/fl*^ mice (n = 7 each). (B) Cell number (top) and percentage of CD45 + cells (bottom) of TCR^−^ IELs in the large intestine lamina propria comparing *C10orf99*^*fl/fl*^ vs. *Villin*^*Cre*^*C10orf99*^*fl/fl*^ mice (n = 14 (WT), 13 (CKO) for cell number; n = 23 (WT), 21 (CKO) for percentage). Data are shown as mean ± SEM. Populations compared using Student’s *t*-test. Each dot represents one mouse.

To determine whether epithelium-derived C10ORF99 plays a role in mediating the localization of IELs in the large intestine epithelium, we crossed our newly developed *C10orf99 cKO* mice with *Villin*^*Cre*^ mice, with the intention of deleting *C10orf99* from the large intestine epithelial cells. We confirmed that *Villin*^*Cre*^*C10orf99*^*fl/fl*^ mice have the intended deletion of *C10orf99* locus only in the intestinal epithelium (Fig. S3). While *Villin*^*Cre*^ is expressed in the epithelium of both small and large intestines^31^, C10ORF99 is not expressed in the small intestine epithelium^21,25^. Therefore, using *Villin*^*Cre*^ mice can achieve the selective removal of C10ORF99 expression in the large intestinal epithelium. When we looked for IEL populations in *C10orf99*^*fl/fl*^ and *Villin*^*Cre*^*C10orf99*^*fl/fl*^ mice, we observed similar trends in *Villin*^*Cre*^*C10orf99*^*fl/fl*^ *mice* to global *C10orf99* KO mice, with severely reduced TCRγδ^+^ nIELs, TCRαβ^+^nIELs, and CD8αβ^+^TCRαβ^+^ pIELs in the large intestine (Fig. 4A). In addition, we saw no significant changes in CD4^+^TCRαβ^+^pIELs and TCR^−^ IEL populations (Fig. 4A) as shown in global *C10orf99* KO mice. We also analyzed lamina propria T cells between *C10orf99*^*fl/fl*^ and *Villin*^*Cre*^*C10orf99*^*fl/fl*^ mice (Fig. 4B). We found that there is a marginal, but statistically significant reduction in GPR15^+^ T cells in the lamina propria when epithelium-derived C10ORF99 is absent (Fig. 4B), but not to the level in the global *C10orf99* KO mice^21^, indicating that epithelium-derived C10ORF99 may further guide GPR15^+^ T cells after their extravasation through blood endothelium or help them stay in the lamina propria.

## Discussion

In this study, we show that C10ORF99-GPR15, previously known as a lamina propria axis^20,21^, is also required for TCR + natural IEL localization to the large intestine epithelium, with epithelium-derived C10ORF99 as a required source. This indicates that lymphocyte localization in the large intestine is separately controlled, even within the epithelium as well as the lamina propria, from that in the small intestine. We showed that with *Gpr15* deficiency, there is a significant reduction in the population of TCR^+^ nIELs in the large intestine (Fig. 1B). In a reciprocal experiment, we observed a similar phenotype in *C10orf99*-deficient mice (Fig. 2A), indicating that the C10ORF99-GPR15 signaling axis is required for the proper localization of these TCR^+^ nIELs. Selective deletion of C10ORF99 in the epithelium showed the same phenotype as whole body deletion of C10ORF99 (Fig. 4), indicating that epithelium-derived C10ORF99 is required for nIEL localization in the large intestine epithelium. While the axis is shown to be selective to nIELs, the discrepancy in the CD8αβ^+^TCRαβ^+^ pIEL phenotype between *Gpr15* KO mice and *C10orf99* KO mice, where only *C10orf99* loss caused a significant reduction (Fig. 1B and 2A), likely reflects suboptimal pIEL development in our mice housed in specific pathogen-free (SPF) settings. This interpretation is supported by low variable levels of CD8αβ^+^TCRαβ^+^ pIELs (Fig. 1B and 2A).

Lymphocytes circulate through blood vessels and enter non-lymphoid tissues, such as the large intestine, by sequentially engaging selectins, integrins, and G protein-coupled receptors at the endothelium^32^. While C10ORF99 is expressed in the blood endothelium of the large intestine, it is also expressed in other cell types of the large intestine, such as the epithelial cells^21^. While the relative contribution of C10ORF99 expressed in the endothelium of the large intestine still needs to be assessed, our results with epithelium-specific deletion of *C10orf99* indicate that TCR^+^ nIEL precursors may still require guidance from the C10ORF99-GPR15 pathway after the extravasation to migrate from the endothelium to the epithelium, or to remain within the epithelium after reaching it (Fig. 4A). Interestingly, a reduction in GPR15^+^ T cells in the lamina propria in the absence of epithelium-derived C10ORF99 (Fig. 4B) supports the notion that C10ORF99 functions additionally on T cells after their extravasation, probably by helping further migration or retention in the lamina propria. The use of homing receptor pairs for retention in the epithelium has been demonstrated, with the requirement of the CCL25-CCR9 pathway for IEL retention in the small intestine^29^. The function of the C10ORF99-GPR15 pathway in IEL retention is an important area for future investigation, as blocking this pathway for therapeutic purposes in humans is likely to affect this process. In addition, the failure to properly localize IEL precursors may also affect their differentiation, as shown with CCR9^19^, but it remains to be tested whether the C10ORF99-GPR15 axis has an effect on IEL differentiation in the large intestine.

Our findings have direct implications for therapeutic strategies to target the C10ORF99-GPR15 axis in inflammatory bowel disease. In mice housed in a laboratory setting, the C10ORF99-GPR15 pathway is preferentially required for Treg localization to the large intestine lamina propria, a situation that likely mirrors early life in humans, when Tregs dominate the colonic lamina propria. However, in adult humans, GPR15 + effector T cells accumulate likely due to increased environmental exposure^33^, with a reversal of the GPR15 expression pattern that we and others have shown in human samples^20,24^. On this basis, blocking GPR15 can be proposed as a therapeutic strategy for IBD to limit pathogenic effector T cell recruitment to the colonic lamina propria. Our new findings complicate this prediction. Because the C10ORF99-GPR15 pathway is also required for the localization of TCR + nIELs in the colon epithelium, GPR15 blockade is likely to simultaneously deplete nIELs with protective functions, such as TCRγδ+ nIELs or CD8αα+TCRαβ+ nIELs^34,35^. Therefore, the therapeutic benefits of reducing recruitment of pathogenic T cells may be offset by the loss of protective natural IELs in the colon epithelium.

While we previously confirmed that C10ORF99 is the only ligand of GPR15 in the large intestine^21^, we and others have also shown that C10ORF99 has additional vital functions in regulating epithelial biology that are independent of GPR15^21,36^. Even though the receptors mediating these effects remain unknown, we confirmed that the IEL phenotype is GPR15-dependent (Fig. 2B), distinguishing it from the GPR15-independent function of C10ORF99 in epithelial cells described previously^21^.

Several questions remain unanswered. First, whether the C10ORF99-GPR15 pathway mediates recruitment, retention, or both remains unresolved. This is important for predicting the effect of therapeutic blockade. Second, the function of C10ORF99 produced by endothelial cells needs to be tested, considering the partial phenotype in the lamina propria of *Villin*^*Cre*^*C10orf99*^*fl/fl*^ mice (Fig. 4B). Third, the functional consequences of nIEL loss in the large intestine remain to be tested. Finally, an inconsistent pIEL phenotype may require analyzing pIELs in conditions that favor full development, such as pet-store or wild mice^37,38^. A thorough investigation of these questions will be required in the future to understand the complexity of epithelium-related immune homeostasis.

## Methods

### Ethics

All animal studies were performed according to the protocol approved by the Institutional Animal Care and Usage Committee (IACUC) of Thomas Jefferson University.

### Mice

All mice used were on a C57BL/6 background, with 6–12 weeks of age. Wild-type C57BL/6 were obtained from Jackson Laboratory. *Gpr15*^*gfp/gfp*^ mice were described previously^20^. *C10orf99* KO mice were described previously^21^. C10orf99 cKO mice were created using CRISPR and homologous recombination on mouse embryos^39^. Double-stranded breaks were generated by electroporating two SgRNAs (5’-AGGATGAGGTTGTCGAggGG-3’ and 5’-GAAGCCCCAGTATCCCTGAG-3’) and the CAS9 protein into mouse embryos at the one-cell stage. Repair templates in single-stranded DNA format were electroporated as well. LoxN sites were added before exon 2 using guide RNA 5’-AGGATGAGGTTGTCGAggGG-3’ and repair template 5’-ATGAGAAAAGAAATACTCAGAGGGCACAGTGACCTCCAAGATTTATTTTGTCTCTGGAAAGCCACACAGCATAGGCTACCTCTTGCCTCCcATAACTTCGTATAGTATACCTTATACGAAGTTATcTcgaCAACCTCATCCTGATTTTGTTGTCGTTGGAG-3’ and after exon 3 using guide RNA 5’-GAAGCCCCAGTATCCCTGAG-3’ and repair template 5’-ACCCCCTGCCCTAGGAGCTGCTCCCCTTCTCTAAGGACCAGCTGTGCCCGACTATTACTTACTCATATTAACTCTCTTGTCCCCTCCCCTcATAACTTCGTATAAGGTATACTATACGAAGTTATaGGGATACTGGGGCTTCCATCATGTGCTTTTCATGT-3’. Genotyping for presence of pre exon 2 LoxN site was done using the primers 5’-TCTCCACAGGGCTAAGGTGT-3’ and 5’-ACAACCACCTCCAACGACAA-3’. Post exon 3 LoxN site presence was confirmed using primers 5’-ACAAAGCCCATCCCATCCTG-3’ and 5’-CCAGCTGTGCCCGACTATTA-3’. Genotyping to test for a C10orf99 deletion was done using primers 5’-TCTCCACAGGGCTAAGGTGT-3’ and 5’-CCAGCTGTGCCCGACTATTA-3’. For the analysis, mice were euthanized by CO2 gas or by exsanguination/perfusion according to the IACUC-approved animal protocol. Experimental mice were co-housed for at least 2 weeks before the analysis. Sex was not considered in this study design as preliminary data did not suggest differences in phenotype based on sex.

### Cell Preparation from the intestines

Large intestine lamina propria immune cells were prepared as previously described^33^. For IEL prep, supernatant was collected after DTT and EDTA washes in the LP prep as previously described^40^. Cells were analyzed using a BD Fortessa, BD Symphony, or Cytek Aurora spectral analyzer. To examine deletion of the *C10orf99 locus* in epithelial and other cells of *Villin*^*Cre*^*C10orf99*^*fl/fl*^ mice, we magnetically removed CD45 + cells from the supernatant after DTT and EDTA washes to prepare epithelial cells and removed EpCAM + cells from enzyme-digested large intestine to prepare non-epithelial cells. Genomic DNA for PCR was subsequently prepared using the SPINeasy DNA kit for tissue (MP Biochemicals).

### Antibodies used

BV421-CD8α (1:400 Dilution; Clone 53–6.7 Biolegend 100737), Brilliant Violet 605-NK1.1 (1:400 Dilution; Clone PK136, Biolegend 108739), PE-Cy7-EpCAM (1:4000 Dilution; Clone G8.8, Biolegend 118215), APC/Fire750-TCRb (1:400 Dilution; Clone H57–597, Biolegend 109246), PE-FoxP3 (1:1:200 Dilution; Clone FJK-16 s, Thermo-fisher 12-5773-82), Brilliant Violet 650-CD45 (1:4000 Dilution; Clone 30-F11, Biolegend 103151), Alexa-Fluor700-I-A/I-E (1:1250 Dilution; Clone M5/114.15.2, Biolegend 107621), PerCPCy5.5-TCRγδ (1:400 Dilution; Clone GL3, Biolegend 118117), RB780-CD8β (1:400 Dilution; Clone YTS156.7.7, BD Biosciences 569224), BV785-CD4 (1:400 Dilution; Clone RM4–5, Biolegend 100453), CD19-BUV661 (1:400 Dilution; Clone 1D3, Invitrogen 376-0193-82), BV711-NKp46 (1:400 Dilution; Clone 29A1.4, Biolegend 137621), PE-Dazzle-CD8α (1:400 Dilution; Clone 53–6.7 Biolegend 100761), BV421-EpCAM (1:4000 Dilution; Clone G8.8, Biolegend 118225), PE-Cy7-NKp46 (1:400 Dilution; Clone 29A1.4, Biolegend 137617), FITC-TCRγδ (1:400 Dilution; Clone GL3, Biolegend 118105), BUV805-CD4 (1:400 Dilution; Clone GK1.5, BD Biosciences 612900), Biotin-mGPR15 (1:20 dilution; Clone: S15042I, Biolegend 154602),

## Supplementary Material

1

## Appendix A. Supplementary data

Supplementary data to this article can be found online at https://doi.org/10.1016/j.mucimm.2026.100355.
