# Supplementary material for: Localization of TCR-expressing natural intraepithelial lymphocytes in the colon epithelium depends on GPR15 and epithelium-derived C10ORF99
Source: Mucosal Immunol. Author manuscript; Available in PMC 2026 Sep 1. (PMC13533461; doi:10.1016/j.mucimm.2026.100355)
Supplement: Supplementary file 1 [file NIHMS2202871-supplement-1.docx]

**Supplemental Fig 1. Gating strategy to identify various IEL subsets.**

**Supplemental Fig 2. Small intestinal IELs are not affected by *C10orf99* deficiency.** Cell number and percentage of CD45+ cells of IELs in the small intestine epithelium comparing wild-type and *C10orf99* KO (pink) mice (n= 8 (WT), 6 (KO)). Data are shown as mean ±SEM. Populations compared using Student’s t-test. Each dot represents one mouse.

**Supplemental Fig 3. Deletion of the *C10orf99* gene is specific in the large intestine epithelium of *Villin^Cre^C10orf99^fl/fl^* mice. (A)** PCR strategy to detect WT alleles (*C10orf99^fl/fl^*) and deleted alleles (*C10orf99^d^*). **(B)** Genomic DNA was prepared from the epithelium of the large intestine (Epi: CD45^+^ cells removed) and the rest of the large intestine tissue (Non-epi: EpCAM^+^ cells removed). PCR was performed as shown in panel A.
